# Unrestricted kinematic alignment in varus total knee arthroplasty outperforms mechanical alignment in CPAK I phenotype, but yields comparable outcomes in CPAK IV: A retrospective analysis from the FP‐UCBM Knee Study Group

**DOI:** 10.1002/ksa.70084

**Published:** 2025-10-06

**Authors:** Edoardo Franceschetti, Giancarlo Giurazza, Stefano Campi, Michael T. Hirschmann, Kristian Samuelsson, Andrea Tanzilli, Pietro Gregori, Michele Paciotti, Biagio Zampogna, Rocco Papalia

**Affiliations:** ^1^ Fondazione Policlinico Universitario Campus Bio‐Medico Roma Italy; ^2^ Department of Medicine and Surgery, Research Unit of Orthopaedic and Trauma Surgery Università Campus Bio‐Medico di Roma Roma Italy; ^3^ Department of Orthopaedic Surgery and Traumatology Kantonsspital Baselland (Bruderholz, Liestal, Laufen) Bruderholz Switzerland; ^4^ University of Basel Basel Switzerland; ^5^ Department of Orthopaedics, Institute of Clinical Sciences, Sahlgrenska Academy University of Gothenburg Gothenburg Sweden; ^6^ Department of Orthopaedics Sahlgrenska University Hospital Mölndal Sweden

**Keywords:** coronal plane alignment of the knee, CPAK, cruciate‐retaining total knee arthroplasty, kinematic alignment, knee phenotypes, mechanical alignment

## Abstract

**Purpose:**

To compare clinical outcomes in varus‐aligned patients undergoing cruciate‐retaining total knee arthroplasty (TKA) with mechanical alignment (MA) versus unrestricted kinematic alignment (KA). The hypothesis was that KA would yield superior outcomes, and that preserving joint line obliquity—regardless of alignment technique—would be associated with better results.

**Methods:**

A retrospective analysis of prospectively collected data from 140 KA and 209 MA TKA cases was performed. Inclusion criteria were: end‐stage varus osteoarthritis (aHKA < 178°), MA or unrestricted KA TKA and ≥ 1‐year follow‐up. Exclusion criteria included prior major surgery (osteotomies, fractures) on the affected limb, inadequate preoperative full‐length radiographs, or post‐operative complications unrelated to alignment strategy. Patients were categorised by CPAK phenotype (I, IV and VII) based on the joint line obliquity. Clinical outcomes at 1‐year follow‐up were assessed using the Knee Society Score (KSS) pt.1 and 2, Oxford Knee Score (OKS), SF‐12, and Forgotten Joint Score (FJS). ANOVA was used to compare results of MA and KA in the overall varus population, and in CPAK subgroups. Statistical significance was set at *p* < 0.05.

**Results:**

KA led to significantly higher KSS pt.1 (84.6 ± 15.3 vs. 73.9 ± 18.9; *p* < 0.001) and FJS (90.5 ± 15.3 vs. 80.4 ± 15.8; *p* < 0.001) than MA. In CPAK I patients, KA outperformed MA in KSS pt.1 (83.5 ± 16.2 vs. 74.9 ± 19.1; *p* < 0.001) and FJS (89.8 ± 15.5 vs. 80.7 ± 17.8; *p* < 0.001). No differences were found between KA and MA in CPAK IV patients (*p* > 0.05). KSS pt.1 (80.1 ± 13.8) and FJS (86.5 ± 18.1) achieved with MA in CPAK IV were significantly higher than in both the overall varus aHKA group and CPAK I patients treated with MA (*p* < 0.05).

**Conclusions:**

Cruciate‐retaining unrestricted KA yields better clinical outcomes compared to MA in varus aHKA patients, and in the CPAK I subgroup. In CPAK IV, preserving joint line obliquity leads to similar outcomes with both MA and KA.

**Level of Evidence:**

Level IV.

**Abbreviations**
aHKA: anatomical hip‐knee‐ankle angle
BMI: body mass index
CPAK: coronal plane alignment of the knee
FJS: Forgotten Joint Score
ICC: intra‐class correlation
JLO: joint line obliquity
KA: kinematic alignment
KSS: Knee Society Score
LDFA: lateral distal femoral angle
MA: mechanical alignment
MPTA: Medial Proximal Tibial Angle
OKS: Oxford Knee Score
PACS: Picture‐Archiving Communication System
ROM: range of motion
SD: standard deviation
SF12: Short Form Survey 12
TKA: total knee arthroplasty

## INTRODUCTION

Despite the growing rate of total knee arthroplasties (TKAs) [7], patient dissatisfaction rates following the procedure still reach as high as 20% [3, 8, 32], with even higher rates of residual symptoms [33]. This may be related to the gold standard alignment technique, Mechanical Alignment (MA) being a “one‐size‐fits‐all” approach, which fails to accommodate the significant natural variations in coronal alignment among individuals [21, 22, 23, 24, 25, 26].

The pursuit of nuanced methods for coronal alignment categorisation—aiming to accommodate the heterogeneity of knee phenotypes within the population—led to the identification of nine Coronal Plane Alignment of the Knee (CPAK) phenotypes proposed by MacDessi et al. [18, 27, 31].

Balancing varus phenotypes with MA may pose a substantial technical challenge [31] and patients with preoperative varus arithmetical hip‐knee‐ankle angle (aHKA) (CPAK I, IV and VII) reach significantly lower post‐operative outcomes at 1‐year follow up after MA TKA compared to those with neutral and valgus aHKA [11]. As such, the optimal alignment strategy for varus aHKA patients remains uncertain, and the influence of joint line orientation in this context still largely unexplored.

The main purpose of the current study was to compare one‐year postoperative outcomes after MA and unrestricted Kinematic Alignment (KA) TKA in patients with preoperative varus aHKA. The hypothesis was that KA would result in superior clinical outcomes, and that the preservation of joint line orientation—regardless of the alignment strategy—would be associated with improved results.

## MATERIALS AND METHODS

### Study design and participants

**Figure 1:**
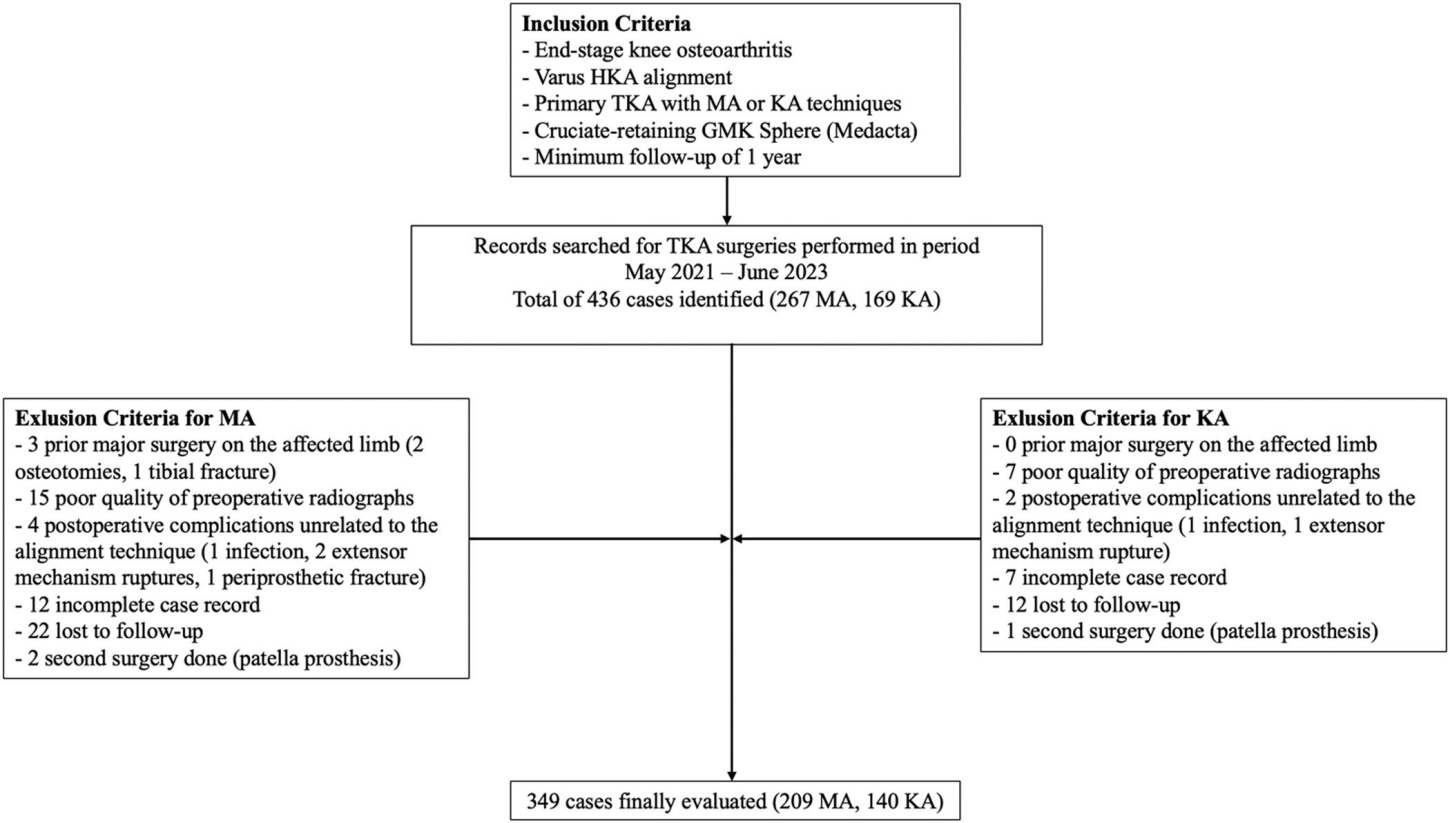
Flowchart depicting inclusion and exclusion criteria. HKA, hip‐knee‐ankle; KA, kinematic alignment; MA, mechanical alignment; TKA, total knee arthroplasty.

Institutional review board approval (IRB No. 32.19 OSS) was granted for this retrospective cohort study, which was conducted in accordance with the Declaration of Helsinki. Written consent was obtained from all included participants. End‐stage knee osteoarthritic patients (Grade IV Kellgren–Lawrence) with varus aHKA undergoing TKA at our Institution (Fondazione Policlinico Universitario Campus Bio‐Medico) with either mechanical or unrestricted kinematic alignment technique and a minimum 1‐year follow‐up, were deemed eligible for inclusion. Exclusion criteria included a history of major surgery on the affected limb (e.g., osteotomies or fractures), absence or poor quality of full‐length anteroposterior preoperative radiographs, and events unrelated to alignment strategy and influencing postoperative outcomes (e.g., postoperative periprosthetic fractures, or revisions for infection or loosening). A flowchart depicting patient inclusion and exclusion criteria is shown in Figure 1.

The study group consisted of 132 consecutive patients (140 knees) who underwent KA TKA [4, 13, 15, 16] between June 2022 and June 2023. The control group consisted of 200 consecutive patients (209 knees) who underwent MA TKA [12] between May 2021 and May 2022. The same implant design (GMK Sphere, Medacta) with PCL preservation was used in both groups. None of the patients had their patella resurfaced at the time of index surgery.

### Radiographic analysis

Preoperative standing full‐length radiographs [17] were used to measure the medial proximal tibial angle (MPTA), the lateral distal femoral angle (LDFA). The arithmetic hip‐knee‐ankle angle (aHKA) [31] was calculated as the difference between MPTA and LDFA and an aHKA < 2° defined varus. Within this category, patients were divided into CPAK phenotypes I, IV and VII, based on the joint line obliquity (JLO: MPTA + LDFA).

All measurements were conducted by a single observer (A.T.) using the tools available in a picture‐archiving communication system (PACS) and recorded to the nearest 0.1°. Two separate measurements were taken six weeks apart, with an ICC of 0.92. Additionally, a second researcher (B.Z.) repeated the measurements on 30 patients to determine inter‐observer reliability (ICC of 0.88).

### Clinical evaluation

Data collection included immediate preoperative and 1‐year postoperative Knee Society Score (KSS) pt. 1 (Objective) and pt. 2 (Functional) [28], Oxford Knee Score (OKS) and Short Form Survey 12 (SF‐12). Additionally, for the postoperative period only, the Forgotten Joint Score (FJS) was assessed [12].

### Data analyses

Descriptive statistics were performed to describe mean, range and standard deviation (SD) for all variables. All data analyses were performed using STATA 18 Software (StataCorp LLC, Lakeway Drive College Station, Texas, USA). Repeated‐measures analysis of variance (ANOVA) was used to evaluate the effects of MA and KA on KSS, OKS, SF‐12, and FJS scores. Comparisons were made between MA and KA in the overall varus population, as well as between MA and KA within CPAK type I and CPAK type IV subgroups. Additionally, KSS pt.1 and FJS obtained in CPAK type IV with MA were compared to those obtained with MA in CPAK I and in the overall varus population. The significance level was set at *P* < 0.05. *Post hoc* power analysis, conducted using G*Power software, indicated that the study sample had approximately 99% power to detect a mean difference of 10.1 ± 15.6 points in FJS (two‐tailed *t*‐test, *α* = 0.05) in the varus knees in the KA group versus the MA group.

## RESULTS

No statistically significant differences were found between the two groups in terms of preoperative demographic characteristics and preoperative clinical scores (Table 1).

**Table 1:** Preoperative demographic characteristics and clinical scores in the control (MA) and the study (KA) groups.

Demographics	Control group (*n* = 209)	Study group (*n *= 140)	*p* value
	Mean (SD)	Mean (SD)	
Age (year)	70.3 (7.1)	70.1 (7.0)	0.809
Gender (Female, *n* (%))	124 (59)	83 (59.3)	0.862
Height (cm)	163.7 (9.1)	164.3 (9.2)	0.579
BMI	29.3 (4.5)	29.2 (6.4)	0.743
aHKA	176.2 (4.5)	175.6 (5.4)	0.319
mHKA	170.1 (4.1)	170.2 (4.2)	0.839
MPTA	85.5 (3.1)	85.2 (3.4)	0.441
LDFA	89.3 (3.3)	89.8 (3.1)	0.183
KSS pt.1	38.9 (14.2)	39.1 (13.9)	0.904
KSS pt.2	47.9 (16.9)	47.4 (16.8)	0.801
OKS	19.7 (6.8)	19.4 (7.0)	0.714
SF12 physical	32.3 (7.1)	32.8 (7.4)	0.561
SF12 mental	45.9 (9.0)	45.8 (9.5)	0.927

Abbreviations: aHKA, arithmetic hip‐knee‐ankle angle; BMI, body mass index; KA, kinematic alignment; KSS, Knee Society Score; LDFA, lateral distal femoral angle; MA, mechanical alignment; MPTA, medial proximal tibial angle; OKS, Oxford Knee Score; SD, standard deviation; SF12, Short Form Survey 12.

The analysis of postoperative clinical outcomes at 1 year after surgery demonstrated statistically significant superior results for KA compared to MA in terms of KSS pt.1 (84.6 ± 15.3 vs. 73.9 ± 18.9; *p* < 0.001) and FJS (90.5 ± 15.3 vs. 80.4 ± 15.8; *p* < 0.001), as shown in Table 2.

**Table 2:** Postoperative outcomes in the control (MA) vs. the study (KA) group.

Score	Alignment		*p* value
	Mechanical	Kinematic	
KSS pt.1	73.9 (18.9)	84.6 (15.3)	**<0.001**
KSS pt.2	89.9 (17.9)	89.4 (18.0)	0.647
OKS	41.5 (7.1)	41.3 (7.8)	0.498
SF12 Physical	50.4 (7.0)	50.2 (8.4)	0.775
SF12 Mental	54.9 (7.7)	53.8 (8.9)	0.145
FJS	80.4 (15.8)	90.5 (15.3)	**<0.001**

Abbreviations: FJS, Forgotten Joint Score; KSS, Knee Society Score; OKS, Oxford Knee Score; SF12, Short Form Survey 12.

**Figure 2:**
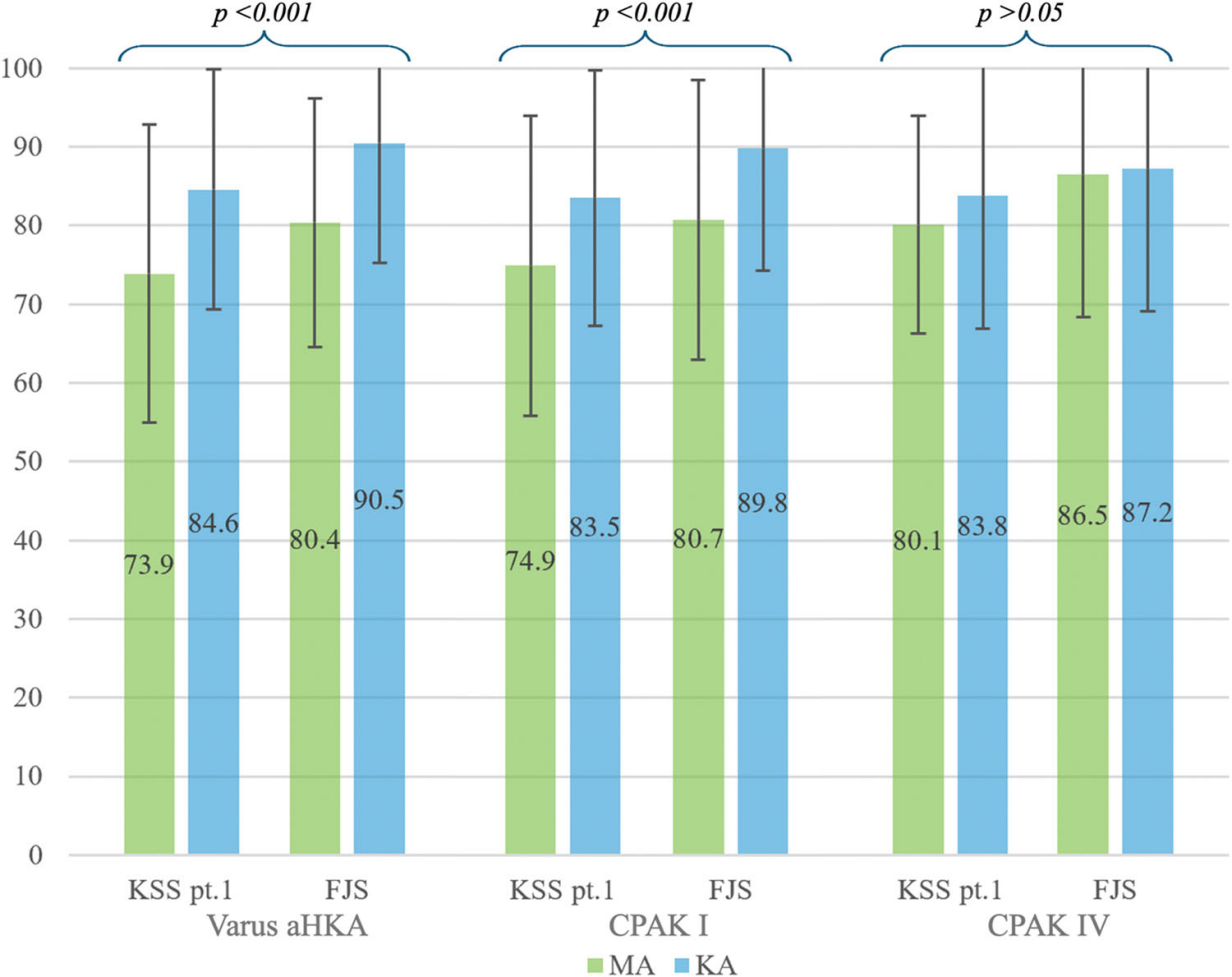
Postoperative outcomes in the control (MA) vs. the study (KA) group in overall varus aHKA, CPAK I and CPAK IV. aHKA, arithmetic hip‐knee‐ankle angle; CPAK, coronal plane alignment of the knee; FJS, Forgotten Joint Score; KA, kinematic alignment; KSS, Knee Society Score; MA, mechanical alignment.

Due to the limited representation of CPAK type VII (11 MA and 6 KA), a direct comparison between the study and control groups for individual CPAK phenotypes was only feasible for Types I and IV (Table 3). KA resulted in statistically significant superior results compared to MA in the CPAK I phenotype in the KSS pt. 1 (83.5 ± 16.2 vs. 74.9 ± 19.1; *p* < 0.001) and FJS (89.8 ± 15.5 vs. 80.7 ± 17.8; *p* < 0.001). No statistically significant differences were found between KA and MA in any post‐operative outcome in the CPAK IV phenotype (*p* > 0.05) (Figure 2). Notably, the FJS (86.5 ± 18.1) and the KSS pt.1 (80.1 ± 13.8) achieved with MA in CPAK IV patients are significantly higher than the average values observed both in the overall varus aHKA group (FJS 80.4 ± 15.8, KSS pt.1 73.9 ± 18.9; *p* < 0.05) and in CPAK I patients (FJS 80.7 ± 17.8, KSS pt.1 74.9 ± 19.1; *p* < 0.05) treated with the same technique.

**Table 3:** Postoperative outcomes in the control (MA) vs the study (KA) group in the CPAK I and IV phenotypes.

	Score	Alignment		*p* value
		Mechanical (*n* = 132)	Kinematic (*n* = 91)	
CPAK I	KSS pt.1	74.9 (19.1)	83.5 (16.2)	**<0.001**
	KSS pt.2	87.9 (18.4)	87.1 (18.3)	0.749
	OKS	40.3 (7.9)	41.5 (8.4)	0.284
	SF12 physical	50.6 (7.4)	49.3 (11.7)	0.350
	SF12 mental	53.2 (8.0)	52.7 (10.3)	0.697
	FJS	80.7 (17.8)	89.8 (15.5)	**<0.001**

		**Alignment**		***p*‐Value**
		Mechanical (*n* = 66)	Kinematic (*n* = 43)	
CPAK IV	KSS pt.1	80.1 (13.8)	83.8 (16.9)	0.234
	KSS pt.2	89.2 (9.1)	88.2 (17.2)	0.727
	OKS	42.1 (6.1)	41.5 (8.2)	0.682
	SF12 physical	51.3 (4.4)	49.7 (7.7)	0.221
	SF12 mental	53.4 (8.3)	52.7 (10.1)	0.706
	FJS	86.5 (18.1)	87.2 (18.1)	0.844

Abbreviations: CPAK, coronal plane alignment of the knee; FJS, Forgotten Joint Score; KSS, Knee Society Score; OKS, Oxford Knee Score; SF12, Short Form Survey 12.

## DISCUSSION

The main finding of the current study was that KA TKA yields higher KSS pt. 1 and FJS compared with MA TKA in patients with preoperative varus aHKA. Subgroup analysis revealed that the benefits of KA over MA were not consistent across the two predominant varus CPAK phenotypes: in CPAK type I, KA led to significantly greater improvements in KSS pt. 1 and FJS; however, no significant differences were observed between KA and MA in CPAK type IV. This study is among the few that compare MA and KA in a varus population and the first to employ an unrestricted KA technique and to specifically focus on preoperative varus aHKA and CPAK phenotypes.

Recent literature has raised concerns regarding the potential for MA to induce gap imbalances that may not be fully correctable with soft‐tissue balancing, leading to postoperative instability [5]. When focusing on varus phenotypes, only 15% of the varus CPAK Types I and IV achieve balance at 10° of flexion after MA TKA, as reported by MacDessi et al. [31]. As a consequence, the more complex soft‐tissue balance of the varus group might offer a potential explanation to the lower clinical outcomes found by Franceschetti et al. [11] in varus aHKA patients undergoing MA TKA.

Our results are in line with those reported by Ettinger et al. [10], who found significant differences in post‐operative FJS and KSS between restricted kinematic alignment (rKA) and MA in patients with varus CPAK phenotypes. Similarly Lin et al. [29] and Elbuluk et al. [9], comparing MA and KA in varus knees, found better post‐operative outcomes and FJS scores in the KA group at 1 year follow‐up.

Compared to varus knees, phenotype‐specific alignment targets might offer fewer advantages for neutral and valgus osteoarthritic knees. Streck et al. [34] found no differences in outcomes across CPAK phenotypes in 158 valgus MA TKA cases, supporting the effectiveness of MA TKA for treating valgus osteoarthritis. Moreover, since MA aims to achieve neutral postoperative HKA, patients with neutral preoperative HKA are likely to respond optimally to this alignment strategy [11]. Therefore, it becomes clear that studies comparing MA and KA without focusing specifically on the varus population tend to underestimate the positive impact of KA in this group [6, 14].

Additionally, our results indicate that the advantage of KA over MA in varus aHKA patients is primarily confined to those with the CPAK I phenotype. In contrast, patients with the CPAK IV phenotype show no statistically significant difference in outcomes between the two alignment strategies. Interestingly, the FJS of 86.5 ± 18.1 and the KSS pt.1 of 80.1 ± 13.8 achieved with MA in CPAK IV patients are substantially higher than the average values observed both in the overall varus aHKA group (FJS 80.4 ± 15.8, KSS pt.1 73.9 ± 18.9) and in CPAK I patients (FJS 80.7 ± 17.8, KSS pt.1 74.9 ± 19.1) treated with the same technique. This finding suggests that preservation of the joint line may be an additional key factor influencing postoperative outcomes. In fact, since both MA and KA preserve the neutral joint line characteristic of CPAK IV, this likely accounts for the comparable balance at 90° of flexion reported by MacDessi et al. [31], as well as the similar clinical outcomes observed in our study. While recent studies have questioned the significance of CPAK phenotype preservation in determining postoperative outcomes [1, 2], additional research exploring the impact of joint line preservation in CPAK V (neutral JLO, neutral aHKA) and CPAK VI (neutral JLO, valgus aHKA) would be valuable to further clarify the complex relationship between preoperative phenotypes and postoperative results.

The present study has limitations to be acknowledged. It is a single‐centre, retrospective cohort study, where the control group underwent surgery prior to the study group. Nevertheless, data collection was uniform, and comparable populations of osteoarthritic patients were evaluated. All surgeries were performed by a small group of experienced surgeons, with the same medial congruent and PCL‐retaining prosthesis, and all patients followed the same postoperative protocol and received the same standard of care. Consequently, we believe that temporal bias and learning curve‐related bias were adequately controlled.

Due to the limited representation of CPAK type VII, a direct comparison between the study and control groups was not feasible for this rare phenotype.

An additional limitation is that our analysis focused exclusively on coronal plane phenotypes. Future studies should also investigate sagittal and rotational phenotypes [20, 35], laxity profiles [19] and extra‐articular deformity patterns [30] to more comprehensively evaluate the three‐dimensional complexity of the knee and its influence on surgical outcomes.

Finally, our analysis was restricted to a one‐year follow‐up. As such, we cannot exclude the possibility that the observed differences in outcomes may diminish over time. Future studies with longer follow‐up are needed to determine whether patients in the MA group simply require a longer recovery period, eventually achieving functional outcomes comparable to those of the KA group.

## CONCLUSIONS

Cruciate‐retaining unrestricted KA yields better clinical outcomes compared to MA in varus aHKA patients, and in the CPAK I subgroup. In CPAK IV, preserving joint line obliquity leads to similar outcomes with both MA and KA. This phenotype‐specific insight can help refine alignment strategies and improve individualised surgical planning.

## AUTHOR CONTRIBUTIONS

Edoardo Franceschetti and Stefano Campi were responsible for data collection and conceptualisation. Giancarlo Giurazza was responsible for writing of the manuscript and qualified as corresponding author. Andrea Tanzilli and Biagio Zampogna were responsible for data analysis. Pietro Gregori supervised data acquisition and analysis. Michele Paciotti was responsible for realisation of Figures and Tables. Michael T Hirschmann, Kristian Samuelsson and Rocco Papalia were responsible for reviewing and critically revise the manuscript. All authors have given final approval of the version to be published.

## CONFLICT OF INTEREST STATEMENT

The authors declare no conflict of interest. Outside the current study, M.T.H. declares being Chief Editor of KSSTA.

## ETHICS STATEMENT

The study was performed in accordance with the ethical standards as laid down in the 1964 Declaration of Helsinki and its later amendments. Institutional review board approval was obtained for this research (IRB no. 32.19 OSS). All patients provided legitimate informed consent.

## Data Availability

The data that support the findings of this study are available from the corresponding author, [G.G.], upon reasonable request.
